# Supplementary material for: Avoidable diet-related deaths and cost-of-illness with culturally optimized modifications in diet: The case of Brazil
Source: PLoS One. 2023 Jul 11;18(7):e0288471. doi: 10.1371/journal.pone.0288471 (PMC10335669; doi:10.1371/journal.pone.0288471)
Supplement: S1 File — (PDF) [file pone.0288471.s001.pdf]

Supplementary Information for the manuscript:

Avoidable diet-related deaths and cost-of-illness with culturally optimized modifications in diet: the case of Brazil.

Eliseu Verly-Jr, Ísis Eloah Machado, Adriana Lúcia Meireles, Eduardo A F Nilson

Table S1 - Sample size and number of foods according to the age-sex group 2

Table S2 - Baseline dietary risk-factors intake and intervention content according to the age-sex group. 2

Table S3 - Mean food intake and upper constraints imposed to the models according to the age-sex group 6

Table S4 - Number of death and uncertainty intervals by dietary risk factor according to the intervention scenario. 15

Table S5 – Number of death and uncertainty intervals by cause according to the intervention scenario. 16

Table S6 – Number of deaths and uncertainty intervals by age-sex group according to the intervention scenario. 17

Table S7 - Cost saved (International Dollars) and uncertainty intervals by cause of death. 18

Table S1- Sample size and number of foods according to the age-sex group

Age-sex group	*n*	*n of foods*
20-30 M	3413	84
30-40 M	3864	84
40-50 M	3661	84
50-60 M	4433	85
60-70 M	3252	83
>70 M	4105	85
20-30 F	3011	83
30-40 F	3613	85
40-50 F	2160	84
50-60 F	2515	83
60-70 F	1628	84
>70 F	2032	83

Table S2- Baseline dietary risk-factors intake and intervention content according to the age-sex group.

Baseline	Fruit (g)		Vegetables (g)		Fibre (g)		Cholesterol (mg)	
	mean	*SD*	*mean*	*SD*	*mean*	*SD*	*mean*	*SD*
20-30 M	51.7	79.2	81.2	51.8	26.6	10.5	336.9	114.0
30-40 M	66.4	89.8	78.9	51.1	20.0	9.1	261.5	100.4
40-50 M	58.1	84.0	92.0	55.1	27.8	10.8	342.0	114.8
50-60 M	71.8	93.3	82.5	52.2	19.6	9.0	246.5	97.5
60-70 M	67.0	90.1	95.9	56.3	27.0	10.6	323.3	111.7
>70 M	81.1	99.1	87.1	53.7	19.4	9.0	241.0	96.4
20-30 F	78.4	97.5	99.9	57.5	26.9	10.6	305.0	108.5
30-40 F	98.9	109.5	92.7	55.3	19.5	9.0	224.4	93.0
40-50 F	85.3	101.7	96.5	56.5	25.2	10.2	270.5	102.1
50-60 F	105.5	113.1	90.2	54.6	19.5	9.0	207.9	89.5
60-70 F	100.3	110.3	94.2	55.8	22.7	9.7	244.8	97.2
>70 F	114.0	117.6	88.4	54.0	18.5	8.8	189.9	85.6

Scenario 1	Fruit (g)		Vegetables (g)		Fibre (g)		Cholesterol (mg)	
	mean	*SD*	*mean*	*SD*	*mean*	*SD*	*mean*	*SD*
20-30 M	76.7	96.4	106.2	59.2	28.6	10.9	321.3	111.3
30-40 M	91.4	105.3	103.9	58.6	22.0	9.6	240.3	96.3
40-50 M	83.1	100.4	117.0	62.2	29.8	11.1	322.0	111.4
50-60 M	96.8	108.4	107.5	59.6	21.6	9.5	229.7	94.1
60-70 M	92.0	105.6	120.9	63.2	29.0	11.0	304.1	108.3
>70 M	106.1	113.4	112.1	60.9	21.4	9.4	221.8	92.5
20-30 F	103.4	112.0	124.9	64.2	28.9	11.0	285.6	104.9
30-40 F	123.9	122.6	117.7	62.4	21.5	9.5	210.2	90.6

40-50 F	110.3	115.7	121.5	63.4	27.2	10.6	254.8	99.1
50-60 F	130.5	125.8	115.2	61.7	21.5	9.5	193.3	86.2
60-70 F	125.3	123.3	120.0	63.0	24.7	10.1	224.9	93.1
>70 F	139.0	129.8	115.2	61.7	20.5	9.2	173.6	81.3

Scenario 2	Fruit (g)		Vegetables (g)		Fibre (g)		Cholesterol (mg)	
	mean	*SD*	*mean*	*SD*	*mean*	*SD*	*mean*	*SD*
20-30 M	101.7	111.0	131.2	65.8	30.6	11.3	285.0	105.1
30-40 M	116.4	118.8	128.9	65.3	24.0	10.0	212.6	90.1
40-50 M	108.1	114.5	142.0	68.5	31.8	11.5	288.9	105.5
50-60 M	121.8	121.6	132.5	66.2	23.6	9.9	198.3	87.7
60-70 M	117.0	119.1	145.9	69.4	31.0	11.4	270.3	102.7
>70 M	131.1	126.1	137.1	67.3	23.4	9.9	192.0	86.1
20-30 F	128.4	124.8	149.9	70.4	30.9	11.3	255.4	101.1
30-40 F	148.9	134.4	142.7	68.7	23.5	9.9	176.8	82.6
40-50 F	135.3	128.1	146.5	69.6	29.2	11.0	220.4	92.2
50-60 F	155.5	137.3	140.2	68.1	23.5	9.9	163.6	79.4
60-70 F	150.3	135.0	144.2	69.0	26.7	10.5	195.0	87.4
>70 F	164.0	141.0	138.4	67.6	22.5	9.7	152.9	76.8

Scenario 3	Fruit (g)		Vegetables (g)		Fibre (g)		Cholesterol (mg)	
	mean	*SD*	*mean*	*SD*	*mean*	*SD*	*mean*	*SD*
20-30 M	126.7	123.9	164.3	73.6	32.6	11.6	282.6	104.6
30-40 M	141.4	131.0	164.3	73.4	26.0	10.4	207.9	89.8
40-50 M	133.1	127.1	174.2	75.7	33.8	11.9	287.2	105.5
50-60 M	146.8	133.4	166.1	73.9	25.6	10.3	193.3	86.7
60-70 M	142.0	131.2	174.4	75.9	33.0	11.7	268.5	101.8
>70 M	156.1	137.6	172.7	75.5	25.4	10.3	187.1	84.9
20-30 F	153.4	136.4	183.4	77.9	32.9	11.7	251.6	98.5
30-40 F	173.9	145.2	171.2	75.2	25.5	10.3	172.5	81.6
40-50 F	160.3	139.4	180.6	77.3	31.2	11.4	217.1	91.5
50-60 F	180.5	148.0	177.3	76.5	25.5	10.3	157.9	78.0
60-70 F	175.3	145.8	181.9	77.5	28.7	10.9	192.9	86.2
>70 F	189.0	151.4	165.6	74.0	24.5	10.1	151.4	76.4

Scenario 4	Fruit (g)		Vegetables (g)		Fibre (g)		Cholesterol (mg)	
	mean	*SD*	*mean*	*SD*	*mean*	*SD*	*mean*	*SD*
20-30 M	126.7	123.9	285.1	87.1	36.9	12.1	257.1	98.9
30-40 M	150.4	131.0	185.5	71.6	26.5	10.4	202.5	86.8
40-50 M	133.1	127.1	296.3	93.9	38.2	12.5	266.9	99.4
50-60 M	146.8	133.4	166.1	72.3	25.6	10.3	180.6	83.9
60-70 M	142.0	131.2	278.5	88.5	36.2	12.0	244.8	94.5
>70 M	156.1	137.6	162.1	73.2	25.4	10.3	177.4	81.4
20-30 F	162.9	136.4	293.4	89.5	37.1	12.2	220.8	89.0
30-40 F	173.9	145.2	168.5	74.4	25.5	10.3	164.8	82.9

40-50 F	160.3	139.4	227.8	85.9	33.7	11.7	188.3	88.5
50-60 F	180.5	148.0	190.3	73.9	26.0	10.3	153.1	78.0
60-70 F	197.3	145.8	270.3	93.2	32.4	11.5	148.5	88.8
>70 F	201.5	151.4	217.3	77.5	25.8	10.2	133.0	76.7

Scenario 5	Fruit (g)		Vegetables (g)		Fibre (g)		Cholesterol (mg)	
	mean	*SD*	*mean*	*SD*	*mean*	*SD*	*mean*	*SD*
20-30 M	198.2	138.7	300.9	95.3	38.4	12.5	235.9	95.4
30-40 M	220.3	153.8	220.9	76.5	28.4	10.6	183.1	84.0
40-50 M	156.1	143.9	318.7	99.4	39.9	12.7	239.4	96.1
50-60 M	228.9	164.2	241.5	86.5	29.0	11.0	165.3	76.8
60-70 M	284.8	169.2	341.5	96.6	41.2	12.7	226.3	93.4
>70 M	229.5	163.1	204.8	82.3	27.5	10.7	168.7	80.7
20-30 F	306.9	184.4	331.1	97.1	40.5	12.8	213.5	88.5
30-40 F	257.7	176.4	214.1	83.9	28.1	10.8	157.1	77.8
40-50 F	286.9	174.7	341.5	101.7	38.3	12.4	174.2	82.0
50-60 F	272.8	181.4	222.2	86.2	28.5	10.9	137.4	72.8
60-70 F	293.0	183.6	298.2	99.8	34.9	12.0	146.3	75.2
>70 F	289.9	186.6	186.0	78.9	27.5	10.7	120.7	68.2

Baseline dietary risk-factors intake and intervention content according to the age-sex group.

Baseline	Sodium (mg)		PUFA (%kcal)		Sat. Fat (%kcal)		Cost (BRL)	Cost (Int$)
	mean	*SD*	*mean*	*SD*	*mean*	*SD*		
20-30 M	2882.9	917.2	7.10	1.51	9.24	1.79	3.99	1.75
30-40 M	2219.1	804.7	6.93	1.49	9.49	1.81	3.39	1.49
40-50 M	2866.5	914.6	7.21	1.52	9.36	1.80	4.10	1.80
50-60 M	2061.2	775.6	6.91	1.48	9.46	1.81	3.14	1.38
60-70 M	2663.3	881.6	7.20	1.52	9.24	1.79	3.78	1.66
>70 M	1973.5	758.9	6.94	1.49	9.46	1.81	2.99	1.31
20-30 F	2593.3	870.0	7.15	1.51	9.22	1.79	3.67	1.61
30-40 F	1868.3	738.4	6.81	1.47	9.36	1.80	2.96	1.30
40-50 F	2343.1	826.9	7.14	1.51	9.22	1.79	3.36	1.47
50-60 F	1795.6	723.9	6.69	1.46	9.25	1.79	2.84	1.25
60-70 F	2105.4	783.8	6.77	1.47	9.25	1.79	3.16	1.39
>70 F	1697.3	703.8	6.31	1.42	9.23	1.79	2.73	1.20

Scenario 1	Sodium (mg)		PUFA (%kcal)		Sat. Fat (%kcal)		Cost (BRL)	Cost (Int$)
	mean	*SD*	*mean*	*SD*	*mean*	*SD*		
20-30 M	2877.1	922.1	7.10	1.51	8.93	1.76	4.03	1.77
30-40 M	2212.4	806.0	6.93	1.49	8.82	1.75	3.42	1.50
40-50 M	2863.6	917.1	7.21	1.52	8.86	1.75	4.13	1.81
50-60 M	2063.3	785.8	6.91	1.48	9.02	1.77	3.20	1.40
60-70 M	2652.6	881.1	7.20	1.52	8.71	1.74	3.80	1.67
>70 M	1959.6	757.2	6.94	1.49	8.81	1.75	2.99	1.31

20-30 F	2585.5	871.4	7.15	1.51	8.65	1.73	3.68	1.61
30-40 F	1855.2	739.4	6.81	1.47	8.98	1.77	2.98	1.31
40-50 F	2338.4	832.6	7.14	1.51	8.82	1.75	3.38	1.48
50-60 F	1786.6	725.0	6.69	1.46	8.85	1.75	2.86	1.25
60-70 F	2101.2	783.6	6.77	1.47	8.57	1.72	3.17	1.39
>70 F	1692.2	711.4	6.31	1.42	8.69	1.71	2.76	1.21

Scenario 2	Sodium (mg)		PUFA (%kcal)		Sat. Fat (%kcal)		Cost (BRL)	Cost (Int$)
	mean	***SD***	***mean***	***SD***	***mean***	***SD***		
20-30 M	2871.3	920.3	7.10	1.51	8.20	1.68	4.15	1.82
30-40 M	2214.7	806.9	6.93	1.49	8.12	1.68	3.58	1.57
40-50 M	2852.2	913.4	7.21	1.52	8.30	1.69	4.22	1.85
50-60 M	2055.1	782.7	6.91	1.48	8.04	1.66	3.30	1.45
60-70 M	2647.3	879.4	7.20	1.52	8.18	1.68	3.86	1.69
>70 M	1967.5	760.2	6.94	1.49	7.99	1.67	3.13	1.37
20-30 F	2590.7	873.2	7.15	1.51	8.17	1.68	3.74	1.64
30-40 F	1855.2	739.4	6.81	1.47	7.94	1.66	3.11	1.36
40-50 F	2338.4	832.6	7.14	1.51	8.12	1.68	3.42	1.50
50-60 F	1792.0	727.2	6.69	1.46	7.89	1.65	2.94	1.29
60-70 F	2099.1	782.8	6.77	1.47	8.08	1.67	3.23	1.42
>70 F	1695.6	712.9	6.31	1.42	8.02	1.66	2.86	1.25

Scenario 3	Sodium (mg)		PUFA (%kcal)		Sat. Fat (%kcal)		Cost (BRL)	Cost (Int$)
	mean	***SD***	***mean***	***SD***	***mean***	***SD***		
20-30 M	2874.2	921.2	7.10	1.51	8.15	1.68	4.35	1.91
30-40 M	2216.9	807.7	6.93	1.49	8.13	1.67	3.73	1.64
40-50 M	2860.8	916.2	7.21	1.52	8.24	1.69	4.43	1.94
50-60 M	2050.9	781.1	6.91	1.48	8.08	1.67	3.47	1.52
60-70 M	2647.3	879.4	7.20	1.52	8.08	1.67	4.11	1.80
>70 M	1967.5	760.2	6.94	1.49	7.97	1.66	3.33	1.46
20-30 F	2582.9	870.5	7.15	1.51	8.14	1.68	4.01	1.76
30-40 F	1862.7	742.3	6.81	1.47	7.95	1.66	3.28	1.44
40-50 F	2336.1	831.7	7.14	1.51	8.16	1.68	3.70	1.62
50-60 F	1790.2	726.4	6.69	1.46	7.86	1.65	3.18	1.39
60-70 F	2101.2	783.6	6.77	1.47	8.07	1.67	3.51	1.54
>70 F	1695.6	712.9	6.31	1.42	7.62	1.62	3.04	1.33

Scenario 4	Sodium (mg)		PUFA (%kcal)		Sat. Fat (%kcal)		Cost (BRL)	Cost (Int$)
	mean	***SD***	***mean***	***SD***	***mean***	***SD***		
20-30 M	2868.4	919.3	8.52	1.65	7.39	1.60	4.40	1.93
30-40 M	2214.7	806.9	8.32	1.63	7.59	1.62	3.59	1.58
40-50 M	2860.8	916.2	8.65	1.66	7.49	1.61	4.50	1.97
50-60 M	2050.9	781.1	8.29	1.63	7.57	1.62	3.27	1.43
60-70 M	2650.0	880.3	8.64	1.66	7.39	1.60	4.12	1.81
>70 M	1963.6	758.7	8.32	1.63	7.57	1.62	3.16	1.39

20-30 F	2582.9	870.5	8.58	1.65	7.37	1.60	4.08	1.79
30-40 F	1866.4	743.8	8.17	1.62	7.49	1.61	3.05	1.34
40-50 F	2336.1	831.7	8.57	1.65	7.37	1.60	3.48	1.53
50-60 F	1788.4	725.7	8.03	1.60	7.40	1.60	3.00	1.31
60-70 F	2099.1	782.8	8.12	1.61	7.40	1.60	3.23	1.42
>70 F	1690.5	710.7	7.57	1.55	7.38	1.60	2.97	1.30

Scenario 5	Sodium (mg)		PUFA (%kcal)		Sat. Fat (%kcal)		Cost (BRL)	Cost (Int$)
	mean	*SD*	*mean*	*SD*	*mean*	*SD*		
20-30 M	2594.6	831.6	8.52	1.65	7.39	1.60	4.40	1.93
30-40 M	1997.2	727.6	8.32	1.63	7.59	1.62	3.67	1.61
40-50 M	2579.9	826.2	8.65	1.66	7.49	1.61	4.47	1.96
50-60 M	1855.1	706.5	8.29	1.63	7.57	1.62	3.75	1.65
60-70 M	2397.0	796.2	8.64	1.66	7.39	1.60	4.63	2.03
>70 M	1776.1	686.3	8.32	1.63	7.57	1.62	3.43	1.51
20-30 F	2334.0	786.6	8.58	1.65	7.37	1.60	4.28	1.88
30-40 F	1681.4	670.1	8.17	1.62	7.49	1.61	3.45	1.51
40-50 F	2108.8	750.8	8.57	1.65	7.37	1.60	4.30	1.89
50-60 F	1616.0	655.8	8.03	1.60	7.40	1.60	3.33	1.46
60-70 F	1894.8	706.6	8.12	1.61	7.40	1.60	3.74	1.64
>70 F	1527.6	642.2	7.57	1.55	7.38	1.60	2.92	1.28

Table S3- Mean food intake and upper constraints imposed to the models according to the age-sex group

	20-30 M		30-40 M		40-50 M		50-60 M	
**Food**	*mean*	*upper (p95)*	*mean*	*upper (p95)*	*mean*	*upper (p95)*	*mean*	*upper (p95)*
avocado	1.08	10.866	1.473	10.866	1.148	10.866	1.262	10.866
pineapple	1.169	7.143	0.889	7.143	0.707	7.143	1.256	7.143
zucchini	0.589	6.259	1.074	6.259	0.809	6.259	1.107	6.259
pumpkin	3.517	13.115	3.962	13.115	4.347	13.115	4.196	13.115
açaí	6.84	85.55	6.503	85.55	7.675	85.55	5.23	85.55
chard	0.004	0.982	0.009	0.982	0.025	0.982	0.029	0.982
acerola	0.066	1.745	0.022	1.745	.	.	0.121	1.745
cress	.	.	0.055	1.794	0.017	1.794	0.027	1.794
celery	0.75	2.352	0.939	2.352	0.873	2.352	0.889	2.352
lettuce	6.474	16.975	7.783	16.975	7.589	16.975	8.272	16.975
garlic	6.163	7.981	4.676	7.981	6.597	7.981	4.614	7.981
aim	0.076	3.587	0.101	3.587	0.128	3.587	0.187	3.587
plum	0.094	2.234	0.206	2.234	0.06	2.234	0.164	2.234
rice	180.491	241.565	126.111	241.565	185.811	241.565	124.113	241.565
banana	13.162	34.86	14.968	34.86	16.138	34.86	17.45	34.86

baroa	0.056	3.958	0.133	3.958	0.093	3.958	0.091	3.958
potato	21.144	45.302	21.321	45.302	20.925	45.302	18.631	45.302
eggplant	0.127	4.104	0.199	4.104	0.255	4.104	0.371	4.104
beet	0.43	4.128	0.805	4.128	0.72	4.128	1.053	4.128
red meat	76.629	104.392	58.802	104.392	83.15	104.392	57.527	104.392
broccoli	0.939	4.328	1.462	4.328	1.347	4.328	1.548	4.328
khaki	0.448	6.102	0.184	6.102	0.259	6.102	0.247	6.102
yam	0.735	10.37	0.924	10.37	1.383	10.37	1.231	10.37
starfruit	0.022	3.261	.	.	0.02	3.261	0.051	3.261
onion	26.551	34.289	20.151	34.289	28.717	34.289	20.493	34.289
carrot	5.302	13.668	6.182	13.668	6.488	13.668	6.745	13.668
chicory	0.037	2.195	0.019	2.195	0.056	2.195	0.03	2.195
chayot	2.812	8.639	3.396	8.639	3.246	8.639	3.599	8.639
coconut	0.353	2.654	0.496	2.654	0.607	2.654	0.469	2.654
cookies	18.168	29.024	17.861	29.024	13.36	29.024	12.971	29.024
kale	0.575	6.102	0.806	6.102	0.941	6.102	1.228	6.102
cauliflower	0.847	4.144	1.46	4.144	1.343	4.144	1.467	4.144
pea	0.191	2.077	0.378	2.077	0.185	2.077	0.299	2.077
spinach	0.045	2.603	0.089	2.603	0.138	2.603	0.057	2.603
manioc flour	14.538	54.388	9.447	54.388	14.101	54.388	7.516	54.388
bean	199.644	268.453	129.932	268.453	215.542	268.453	129.974	268.453
chicken	63.409	89.106	53.218	89.106	61.672	89.106	47.858	89.106
chickpea	0.013	3.683	0.061	3.683	0.024	3.683	0.1	3.683
guava	0.832	11.582	0.968	11.582	0.929	11.582	1.397	11.582
yogurt	4.786	21.134	9.347	21.134	5.573	21.134	8.109	21.134
jilo	0.202	7.637	0.135	7.637	0.186	7.637	0.348	7.637
kiwi	0.022	2.84	0.148	2.84	0.105	2.84	0.051	2.84
orange	7.611	31.753	8.949	31.753	9.713	31.753	9.755	31.753
milk	75.419	131.876	77.115	131.876	64.55	131.876	73.485	131.876
lentil	3.726	37.886	1.83	37.886	2.937	37.886	2.741	37.886
apple	6.661	20.883	10.902	20.883	6.263	20.883	11.53	20.883
papaya	1.948	19.237	5.408	19.237	3.451	19.237	6.079	19.237
cassava	12.351	40.991	11.281	40.991	14.624	40.991	12.001	40.991
mango	3.132	18.919	3.619	18.919	2.486	18.919	4.141	18.919
butter	2.614	5.176	2.551	5.176	2.513	5.176	2.311	5.176
margarine	8.96	13.574	7.142	13.574	9.098	13.574	6.977	13.574
pasta	55.228	75.029	43.514	75.029	50.345	75.029	36.617	75.029
watermelon	3.389	21.728	5.012	21.728	3.46	21.728	6.297	21.728
corn	1.613	8.182	1.451	8.182	1.218	8.182	1.567	8.182
sauce	5.474	9.484	3.536	9.484	4.082	9.484	3.011	9.484
strawberry	0.252	2.963	0.238	2.963	0.226	2.963	0.35	2.963
nuts	0.422	2.612	0.521	2.612	0.476	2.612	0.385	2.612
olive oil	2.11	8.421	2.054	8.421	2.547	8.421	2.666	8.421
egg	22.07	31.234	16.112	31.234	19.813	31.234	15.903	31.234
buns ans rolls	65.908	82.451	47.854	82.451	63.473	82.451	46.829	82.451

peanut	0.193	1.25	0.201	1.25	0.278	1.25	0.154	1.25
fish	25.227	88.875	21.522	88.875	30.168	88.875	18.171	88.875
cucumber	0.362	3.059	0.49	3.059	0.31	3.059	0.593	3.059
pear	0.314	4.61	0.741	4.61	0.888	4.61	1.078	4.61
peach	0.052	6.184	0.22	6.184	0.391	6.184	0.491	6.184
pepper	0.858	2.322	0.827	2.322	1.154	2.322	0.687	2.322
pork	21.483	64.094	14.074	64.094	26.759	64.094	14.632	64.094
processed meat	26.133	32.817	18.142	32.817	24.103	32.817	15.523	32.817
cheese	12.364	25.678	10.678	25.678	12.806	25.678	9.492	25.678
okra	0.695	8.348	0.532	8.348	0.618	8.348	0.878	8.348
cabbage	1.023	7.973	1.266	7.973	1.097	7.973	1.095	7.973
arugula	0.419	5.499	0.605	5.499	0.787	5.499	0.992	5.499
parsley	0.214	0.486	0.205	0.486	0.224	0.486	0.216	0.486
seafood	0.829	7.131	0.684	7.131	1.239	7.131	0.551	7.131
snacks	6.74	14.457	6.342	14.457	4.99	14.457	4.467	14.457
sugar-sweetened beverages	114.065	131.223	76.446	131.223	89.263	131.223	55.319	131.223
sugar	29.648	42.167	26.838	42.167	29.977	42.167	26.966	42.167
sweets	9.503	22.808	11.598	22.808	8.658	22.808	8.622	22.808
tangerine	1.503	20.549	3.508	20.549	1.733	20.549	2.387	20.549
spices	0.489	1.455	0.798	1.455	0.477	1.455	0.548	1.455
tomato	20.255	40.377	19.815	40.377	21.816	40.377	19.832	40.377
grape	0.859	7.234	0.779	7.234	1.033	7.234	1.005	7.234
pod	1.336	3.9	1.351	3.9	1.742	3.9	1.555	3.9
whole rice	2.153	11.246	1.814	11.246	2.444	11.246	2.468	11.246
whole brad	7.494	16.655	5.438	16.655	7.715	16.655	5.255	16.655

Table S3 (cont.) - Mean food intake and upper constraints imposed to the models according to the age-sex group

	**60-70 M**		**>70 M**	
**Food**	***mean***	***upper (p95)***	***mean***	***upper (p95)***
avocado	1.506	10.866	1.321	10.866
pineapple	1.316	7.143	1.018	7.143
zucchini	1.447	6.259	0.927	6.259
pumpkin	4.26	13.115	4.572	13.115
açaí	4.067	85.55	3.509	85.55
chard	0.006	0.982	0.044	0.982
acerola	.	.	0.152	1.745
cress	0.084	1.794	0.044	1.794
celery	0.82	2.352	1.02	2.352
lettuce	8.407	16.975	9.04	16.975
garlic	6.486	7.981	4.624	7.981
aim	0.193	3.587	0.24	3.587

plum	0.129	2.234	0.213	2.234
rice	182.843	241.565	118.245	241.565
banana	17.446	34.86	19.334	34.86
baroa	0.217	3.958	0.17	3.958
potato	20.044	45.302	20.179	45.302
eggplant	0.236	4.104	0.448	4.104
beet	0.575	4.128	0.817	4.128
red meat	77.29	104.392	53.004	104.392
broccoli	1.313	4.328	1.08	4.328
khaki	0.747	6.102	0.924	6.102
yam	1.104	10.37	1.341	10.37
starfruit	0.069	3.261	0.018	3.261
onion	28.478	34.289	20.399	34.289
carrot	6.739	13.668	7.36	13.668
chicory	0.004	2.195	0.02	2.195
chayot	3.413	8.639	4.206	8.639
coconut	0.49	2.654	0.508	2.654
cookies	10.714	29.024	10.76	29.024
kale	1.386	6.102	1.186	6.102
cauliflower	1.388	4.144	1.874	4.144
pea	0.128	2.077	0.244	2.077
spinach	.	.	0.048	2.603
manioc flour	12.717	54.388	7.767	54.388
bean	211.921	268.453	128.339	268.453
chicken	59.962	89.106	46.198	89.106
chickpea	0.121	3.683	0.083	3.683
guava	0.861	11.582	1.879	11.582
yogurt	4.797	21.134	8.108	21.134
jilo	0.82	7.637	0.703	7.637
kiwi	0.047	2.84	0.099	2.84
orange	11.717	31.753	11.536	31.753
milk	63.562	131.876	70.59	131.876
lentil	2.82	37.886	2.39	37.886
apple	7.606	20.883	12.162	20.883
papaya	4.608	19.237	8.732	19.237
cassava	14.433	40.991	11.727	40.991
mango	3.334	18.919	4.554	18.919
butter	2.381	5.176	2.361	5.176
margarine	8.216	13.574	6.898	13.574
pasta	47.672	75.029	34.56	75.029
watermelon	5.815	21.728	7.365	21.728
corn	1.713	8.182	1.746	8.182
sauce	2.956	9.484	2.269	9.484
strawberry	0.051	2.963	0.234	2.963
nuts	0.338	2.612	0.467	2.612

olive oil	2.747	8.421	2.68	8.421
egg	19.228	31.234	15.133	31.234
buns ans rolls	59.02	82.451	46.336	82.451
peanut	0.182	1.25	0.223	1.25
fish	27.949	88.875	20.019	88.875
cucumber	0.537	3.059	0.639	3.059
pear	0.5	4.61	1.07	4.61
peach	0.291	6.184	0.413	6.184
pepper	0.879	2.322	0.644	2.322
pork	23.684	64.094	13.525	64.094
processed meat	18.77	32.817	13.851	32.817
cheese	11.567	25.678	10.371	25.678
okra	0.747	8.348	0.755	8.348
cabbage	1.149	7.973	1.348	7.973
arugula	0.877	5.499	1.028	5.499
parsley	0.205	0.486	0.18	0.486
seafood	0.82	7.131	0.767	7.131
snacks	5.047	14.457	4.028	14.457
sugar-sweetened beverages	64.654	131.223	42.342	131.223
sugar	28.372	42.167	24.928	42.167
sweets	8.347	22.808	7.375	22.808
tangerine	4.027	20.549	2.753	20.549
spices	0.407	1.455	0.474	1.455
tomato	22.87	40.377	21.682	40.377
grape	1.371	7.234	1.89	7.234
pod	1.799	3.9	1.614	3.9
whole rice	3.189	11.246	2.435	11.246
whole brad	7.042	16.655	5.693	16.655

Table S3 (cont.) - Mean food intake and upper constraints imposed to the models according to the age-sex group

	**20-30 F**		**30-40 F**		**40-50 F**		**50-60 F**	
**Food**	***mean***	***upper (p95)***	***mean***	***upper (p95)***	***mean***	***upper (p95)***	***mean***	***upper (p95)***
avocado	1.826	10.866	2.063	10.866	1.763	10.866	2.322	10.866
pineapple	0.421	7.143	1.458	7.143	1.476	7.143	1.965	7.143
zucchini	1.612	6.259	1.414	6.259	1.777	6.259	1.734	6.259
pumpkin	5.594	13.115	5.113	13.115	5.802	13.115	5.782	13.115
açaí	3.688	85.55	3.788	85.55	3.286	85.55	2.192	85.55
chard	0.056	0.982	0.026	0.982	0.006	0.982	0.037	0.982
acerola	0.203	1.745	0.062	1.745	0.028	1.745	.	.
cress	0.098	1.794	0.096	1.794	0.076	1.794	0.105	1.794

celery	0.971	2.352	1.054	2.352	1.002	2.352	1.075	2.352
lettuce	8.551	16.975	9.594	16.975	8.825	16.975	8.483	16.975
garlic	6.648	7.981	4.65	7.981	6.184	7.981	4.611	7.981
aim	0.295	3.587	0.206	3.587	0.403	3.587	0.386	3.587
plum	0.146	2.234	0.368	2.234	0.245	2.234	0.262	2.234
rice	184.191	241.565	111.52	241.565	160.477	241.565	111.063	241.565
banana	19.815	34.86	22.337	34.86	23.52	34.86	26.573	34.86
baroa	.	.	0.054	3.958	0.031	3.958	0.139	3.958
potato	23.857	45.302	22.046	45.302	24.324	45.302	21.478	45.302
eggplant	0.194	4.104	0.249	4.104	0.134	4.104	0.356	4.104
beet	0.595	4.128	1.063	4.128	0.678	4.128	0.664	4.128
red meat	75.599	104.392	51.548	104.392	66.738	104.392	47.564	104.392
broccoli	1.254	4.328	1.67	4.328	0.997	4.328	1.426	4.328
khaki	0.729	6.102	0.923	6.102	0.375	6.102	0.915	6.102
yam	1.563	10.37	1.621	10.37	2.623	10.37	2.429	10.37
starfruit	0.05	3.261	0.021	3.261	.	.	.	.
onion	28.56	34.289	20.484	34.289	26.21	34.289	19.618	34.289
carrot	6.728	13.668	7.966	13.668	7.055	13.668	7.841	13.668
chicory	0.053	2.195	0.115	2.195	0.03	2.195	0.093	2.195
chayot	4.198	8.639	5.155	8.639	4.893	8.639	5.596	8.639
coconut	0.476	2.654	0.568	2.654	0.447	2.654	0.726	2.654
cookies	8.63	29.024	9.791	29.024	8.787	29.024	9.682	29.024
kale	1.366	6.102	1.847	6.102	1.452	6.102	1.957	6.102
cauliflower	1.565	4.144	1.698	4.144	1.56	4.144	1.846	4.144
pea	0.299	2.077	0.229	2.077	0.104	2.077	0.206	2.077
spinach	0.056	2.603	0.049	2.603	0.121	2.603	0.054	2.603
manioc flour	11.768	54.388	7.783	54.388	10.978	54.388	6.661	54.388
bean	209.288	268.453	128.736	268.453	195.296	268.453	124.925	268.453
chicken	52.541	89.106	44.887	89.106	49.507	89.106	45.296	89.106
chickpea	.	.	0.153	3.683	0.083	3.683	0.072	3.683
guava	1.162	11.582	2.479	11.582	1.407	11.582	1.555	11.582
yogurt	4.541	21.134	8.067	21.134	2.921	21.134	7.985	21.134
jilo	1.05	7.637	0.455	7.637	0.632	7.637	0.955	7.637
kiwi	0.076	2.84	0.095	2.84	0.016	2.84	0.181	2.84
orange	15.042	31.753	15.453	31.753	16.385	31.753	19.101	31.753
milk	70.851	131.876	75.541	131.876	73.87	131.876	87.28	131.876
lentil	2.315	37.886	1.663	37.886	3.728	37.886	2.707	37.886
apple	8.692	20.883	12.827	20.883	7.233	20.883	13.337	20.883
papaya	6.831	19.237	12.181	19.237	8.546	19.237	12.591	19.237
cassava	14.905	40.991	11.779	40.991	13.83	40.991	12.505	40.991
mango	4.828	18.919	6.888	18.919	3.664	18.919	6.48	18.919
butter	2.089	5.176	1.986	5.176	1.706	5.176	1.832	5.176
margarine	7.871	13.574	6.194	13.574	6.894	13.574	5.697	13.574
pasta	39.762	75.029	28.231	75.029	31.664	75.029	25.362	75.029
watermelon	4.857	21.728	8.01	21.728	7.88	21.728	7.315	21.728

corn	1.884	8.182	1.144	8.182	0.939	8.182	1.462	8.182
sauce	2.632	9.484	1.886	9.484	1.027	9.484	1.362	9.484
strawberry	0.03	2.963	0.135	2.963	0.155	2.963	0.286	2.963
nuts	0.39	2.612	0.424	2.612	0.354	2.612	0.308	2.612
olive oil	2.968	8.421	3.407	8.421	2.82	8.421	2.901	8.421
egg	18.097	31.234	13.323	31.234	14.908	31.234	11.541	31.234
buns ans rolls	57.376	82.451	43.101	82.451	51.043	82.451	42.142	82.451
peanut	0.07	1.25	0.224	1.25	0.229	1.25	0.149	1.25
fish	29.899	88.875	22.524	88.875	27.501	88.875	17.542	88.875
cucumber	0.407	3.059	0.816	3.059	1.066	3.059	1.051	3.059
pear	1.004	4.61	1.034	4.61	0.782	4.61	0.892	4.61
peach	0.733	6.184	0.683	6.184	0.691	6.184	0.742	6.184
pepper	1.023	2.322	0.835	2.322	0.81	2.322	0.673	2.322
pork	28	64.094	13.516	64.094	26.676	64.094	14.614	64.094
processed meat	18	32.817	11.219	32.817	16.22	32.817	10.359	32.817
cheese	11.674	25.678	10.562	25.678	10.273	25.678	9.898	25.678
okra	1.086	8.348	1.152	8.348	1.474	8.348	1.247	8.348
cabbage	1.827	7.973	1.58	7.973	1.442	7.973	1.857	7.973
arugula	0.947	5.499	1.06	5.499	0.957	5.499	1.011	5.499
parsley	0.236	0.486	0.182	0.486	0.21	0.486	0.215	0.486
seafood	0.782	7.131	0.703	7.131	0.754	7.131	0.355	7.131
snacks	3.431	14.457	3.095	14.457	2.363	14.457	2.683	14.457
sugar-sweetened beverages	50.391	131.223	36.724	131.223	36.539	131.223	24.909	131.223
sugar	28.011	42.167	24.55	42.167	24.773	42.167	23.031	42.167
sweets	7.329	22.808	7.367	22.808	7.607	22.808	7.429	22.808
tangerine	5.672	20.549	4.462	20.549	5.351	20.549	4.69	20.549
spices	0.47	1.455	0.444	1.455	0.559	1.455	0.569	1.455
tomato	22.557	40.377	21.69	40.377	20.097	40.377	18.908	40.377
grape	0.873	7.234	1.883	7.234	0.842	7.234	1.792	7.234
pod	1.746	3.9	1.689	3.9	1.678	3.9	1.969	3.9
whole rice	3.267	11.246	3.338	11.246	3.062	11.246	3.956	11.246
whole brad	6.619	16.655	5.131	16.655	5.144	16.655	5.734	16.655

Table S3 (cont.) - Mean food intake and upper constraints imposed to the models according to the age-sex group

	**60-70 F**		**>70 F**	
**Food**	***mean***	***upper (p95)***	***mean***	***upper (p95)***
avocado	2.297	10.866	1.036	10.866
pineapple	1.953	7.143	1.163	7.143

zucchini	2.085	6.259	1.822	6.259
pumpkin	6.742	13.115	8.046	13.115
açaí	2.331	85.55	3.006	85.55
chard	0.044	0.982	0.124	0.982
acerola	.	.	.	.
cress	0.012	1.794	0.037	1.794
celery	1.593	2.352	1.561	2.352
lettuce	7.212	16.975	7.586	16.975
garlic	6.009	7.981	4.823	7.981
aim	0.248	3.587	0.268	3.587
plum	0.244	2.234	0.467	2.234
rice	139.145	241.565	99.275	241.565
banana	30.159	34.86	32.438	34.86
baroa	0.399	3.958	0.061	3.958
potato	21.78	45.302	23.388	45.302
eggplant	0.316	4.104	0.384	4.104
beet	0.678	4.128	0.75	4.128
red meat	65.74	104.392	47.186	104.392
broccoli	1.135	4.328	1.224	4.328
khaki	0.866	6.102	0.812	6.102
yam	2.174	10.37	1.884	10.37
starfruit	0.046	3.261	.	.
onion	23.919	34.289	18.943	34.289
carrot	9.85	13.668	9.973	13.668
chicory	0.351	2.195	0.263	2.195
chayot	6.248	8.639	6.804	8.639
coconut	0.518	2.654	0.536	2.654
cookies	10.29	29.024	10.437	29.024
kale	1.082	6.102	1.478	6.102
cauliflower	1.43	4.144	1.427	4.144
pea	0.038	2.077	0.283	2.077
spinach	0.097	2.603	0.162	2.603
manioc flour	8.9	54.388	5.705	54.388
bean	164.413	268.453	117.754	268.453
chicken	48.937	89.106	43.977	89.106
chickpea	0.071	3.683	0.192	3.683
guava	0.731	11.582	1.449	11.582
yogurt	6.241	21.134	10.46	21.134
jilo	1.25	7.637	0.679	7.637
kiwi	0.093	2.84	0.112	2.84
orange	20.251	31.753	20.048	31.753
milk	107.603	131.876	127.45	131.876
lentil	2.099	37.886	1.713	37.886
apple	8.393	20.883	13.204	20.883
papaya	12.552	19.237	15.504	19.237

cassava	13.554	40.991	10.029	40.991
mango	5.827	18.919	5.945	18.919
butter	1.769	5.176	1.709	5.176
margarine	6.297	13.574	4.906	13.574
pasta	28.573	75.029	23.655	75.029
watermelon	4.865	21.728	7.548	21.728
corn	1.589	8.182	1.107	8.182
sauce	0.787	9.484	0.846	9.484
strawberry	0.037	2.963	0.094	2.963
nuts	0.282	2.612	0.326	2.612
olive oil	2.609	8.421	2.118	8.421
egg	13.899	31.234	9.731	31.234
buns ans rolls	49.095	82.451	40.066	82.451
peanut	0.112	1.25	0.091	1.25
fish	18.946	88.875	14.536	88.875
cucumber	0.66	3.059	0.484	3.059
pear	0.759	4.61	1.745	4.61
peach	0.578	6.184	0.946	6.184
pepper	0.782	2.322	0.551	2.322
pork	21.406	64.094	11.348	64.094
processed meat	10.238	32.817	7.556	32.817
cheese	9.419	25.678	9.177	25.678
okra	1.447	8.348	1.385	8.348
cabbage	1.297	7.973	1.367	7.973
arugula	0.374	5.499	0.812	5.499
parsley	0.19	0.486	0.179	0.486
seafood	0.394	7.131	0.192	7.131
snacks	1.644	14.457	1.946	14.457
sugar-sweetened beverages	24.708	131.223	19.185	131.223
sugar	26.888	42.167	24.535	42.167
sweets	7.981	22.808	8.191	22.808
tangerine	5.716	20.549	4.649	20.549
spices	0.547	1.455	0.379	1.455
tomato	16.713	40.377	15.182	40.377
grape	1.144	7.234	2.218	7.234
pod	1.832	3.9	1.636	3.9
whole rice	5.242	11.246	3.607	11.246
whole brad	5.486	16.655	5.25	16.655

Table S4- Number of death and uncertainty intervals by dietary risk factor according to the intervention scenario.

	**Scenario 1**			**Scenario 2**			**Scenario 3**			**Scenario 4**			**Scenario 5**		
	Mean	***2.5th***	***97.5th***	***Mean***	***2.5th***	***97.5th***	***Mean***	***2.5th***	***97.5th***	***Mean***	***2.5th***	***97.5th***	***Mean***	***2.5th***	***97.5th***
Diet	12,750	10,178	15,225	23,830	19,241	28,200	32,739	26,354	38,589	37,317	30,468	43,987	57,341	48,573	66,298
Fruit and vegetables	5,749	3,988	7,266	10,782	7,701	13,515	14,963	10,939	18,491	16,445	11,886	20,351	27,852	21,132	33,928
Fibre	4,596	2,600	6,527	8,736	5,073	12,497	12,519	7,206	17,580	14,458	8,346	20,386	17,896	10,404	25,279
Fats	2,095	1,692	2,521	4,802	3,946	5,717	5,775	4,596	7,034	7,033	5,977	8,166	8,557	7,146	10,028
Salt	126	55	198	140	61	220	128	56	202	151	66	238	5,580	2,320	9,010

Table S5 – Number of death and uncertainty intervals by cause according to the intervention scenario.

	Scenario 1			Scenario 2			Scenario 3			Scenario 4			Scenario 5		
	Mean	***2.5th***	***97.5th***	***Mean***	***2.5th***	***97.5th***	***Mean***	***2.5th***	***97.5th***	***Mean***	***2.5th***	***97.5th***	***Mean***	***2.5th***	***97.5th***
Cardiovascular disease	11,971	9,400	14,388	22,682	18,110	27,040	31,008	24,727	36,783	35,282	28,553	42,014	54,132	45,297	63,070
Coronary heart disease	8,574	6,428	10,538	16,384	12,639	19,877	21,483	16,598	25,919	24,849	19,660	29,786	34,022	27,346	39,998
Stroke	3,271	1,863	4,635	6,308	3,717	8,814	9,366	5,699	13,141	10,524	5,969	14,782	17,838	12,103	23,155
Heart failure	0	0	0	0	0	0	0	0	0	0	0	0	411	169	680
Aortic aneurysm	0	0	0	0	0	0	0	0	0	0	0	0	109	44	181
Pulmonary embolism	0	0	0	0	0	0	0	0	0	0	0	0	53	17	109
Hypertensive disease	0	0	0	0	0	0	0	0	0	0	0	0	1,742	722	2,852
Lung cancer	266	109	410	540	229	826	818	335	1,244	877	356	1,345	1,735	697	2,630
Colorectum cancer	318	120	506	636	257	998	932	354	1,468	1,139	446	1,782	1,518	596	2,379

Table S6 – Number of deaths and uncertainty intervals by age-sex group according to the intervention scenario.

	Scenario 1			Scenario 2			Scenario 3			Scenario 4			Scenario 5		
	Mean	***2.5th***	***97.5th***	***Mean***	***2.5th***	***97.5th***	***Mean***	***2.5th***	***97.5th***	***Mean***	***2.5th***	***97.5th***	***Mean***	***2.5th***	***97.5th***
M25-29	24	19	28	48	39	57	69	56	81	74	60	86	125	106	144
M30-34	46	37	54	93	76	110	133	109	157	143	117	168	242	205	279
M35-39	81	65	97	166	136	196	237	193	280	254	208	298	430	365	496
M40-44	141	113	168	288	235	339	410	335	485	440	360	516	744	631	859
M45-49	237	190	283	486	396	572	693	565	818	743	607	871	1,256	1,065	1,450
M50-54	375	301	448	769	627	905	1,095	894	1,293	1,175	960	1,378	1,986	1,685	2,293
M55-59	509	407	607	1,042	850	1,227	1,485	1,212	1,754	1,593	1,302	1,868	2,694	2,285	3,109
M60-64	637	510	760	1,305	1,064	1,536	1,859	1,517	2,195	1,995	1,630	2,339	3,372	2,860	3,892
M65-69	708	567	845	1,451	1,183	1,708	2,067	1,686	2,440	2,217	1,812	2,600	3,749	3,180	4,327
M70-74	738	591	880	1,512	1,233	1,780	2,155	1,758	2,544	2,311	1,888	2,710	3,908	3,315	4,510
M75-79	768	615	916	1,574	1,284	1,853	2,243	1,830	2,648	2,406	1,966	2,821	4,068	3,450	4,695
M80-84	722	578	861	1,479	1,206	1,741	2,107	1,719	2,488	2,260	1,847	2,651	3,821	3,242	4,411
M85+	726	581	865	1,486	1,212	1,750	2,118	1,728	2,500	2,272	1,856	2,664	3,841	3,258	4,433
F25-29	25	19	30	44	34	53	58	46	69	70	57	84	98	82	114
F30-34	54	41	66	96	75	116	127	101	151	154	124	184	215	180	250
F35-39	95	72	117	169	132	204	224	177	266	271	218	322	377	317	439
F40-44	148	111	181	262	204	316	347	274	412	420	337	500	585	491	681
F45-49	231	173	283	410	319	493	542	428	644	656	527	781	914	768	1,063
F50-54	349	262	428	620	483	746	820	647	975	993	798	1,182	1,383	1,162	1,609
F55-59	464	349	569	824	643	993	1,091	861	1,296	1,320	1,061	1,572	1,840	1,545	2,140
F60-64	580	435	711	1,029	802	1,239	1,362	1,075	1,619	1,648	1,324	1,962	2,297	1,929	2,672
F65-69	685	514	839	1,216	948	1,464	1,609	1,269	1,912	1,947	1,564	2,318	2,713	2,278	3,156
F70-74	787	591	964	1,397	1,089	1,682	1,849	1,459	2,197	2,237	1,797	2,663	3,118	2,618	3,626
F75-79	923	694	1,132	1,640	1,279	1,975	2,171	1,712	2,579	2,626	2,110	3,127	3,660	3,074	4,257
F80-84	1,222	918	1,498	2,169	1,691	2,612	2,871	2,265	3,412	3,474	2,791	4,136	4,842	4,066	5,631
F85+	1,232	925	1,510	2,187	1,705	2,634	2,895	2,284	3,439	3,502	2,814	4,170	4,881	4,099	5,677

Table S7- Cost saved (International Dollars) and uncertainty intervals by cause of death.

	Scenario 1			Scenario 2			Scenario 3		
	Mean	*2.5th*	*97.5th*	*Mean*	*2.5th*	*97.5th*	*Mean*	*2.5th*	*97.5th*
Cardiovascular disease	108,974,566	86,628,866	129,271,729	214,229,129	173,725,221	252,321,680	282,529,461	230,098,021	333,797,181
Coronary heart disease	93,357,511	72,137,790	112,587,733	184,399,201	146,089,346	220,035,149	237,323,591	186,136,868	285,339,858
Stroke	15,492,302	9,291,464	21,653,606	30,147,195	18,065,317	41,147,147	44,274,292	27,041,660	60,548,471
Heart failure	0	0	0	0	0	0	0	0	0
Aortic aneurysm	0	0	0	0	0	0	0	0	0
Pulmonary embolism	0	0	0	0	0	0	0	0	0
Rheumatic heart disease	0	0	0	0	0	0	0	0	0
Hypertensive disease	0	0	0	0	0	0	0	0	0
Lung cancer	401,762	172,352	617,042	818,463	350,184	1,268,824	1,250,390	510,050	1,904,721
Colorectum cancer	4,189,400	1,615,936	6,666,249	8,289,073	3,078,960	13,042,077	12,224,368	4,556,602	19,172,298

Table S7 (cont.) - Cost saved (International Dollars) and uncertainty intervals by cause of death.

	Scenario 4			Scenario 5		
	Mean	*2.5th*	*97.5th*	*Mean*	*2.5th*	*97.5th*
Cardiovascular disease	317,750,977	259,805,966	372,910,876	476,712,143	398,194,158	547,757,551
Coronary heart disease	268,208,420	212,894,168	319,599,148	380,235,301	305,795,767	442,610,136
Stroke	49,354,131	29,611,048	69,017,984	86,019,424	59,283,019	111,048,623
Heart failure	0	0	0	5,451,832	2,283,506	9,001,995
Aortic aneurysm	0	0	0	2,431,744	1,031,155	4,090,183
Pulmonary embolism	0	0	0	148,749	46,195	298,657
Rheumatic heart disease	0	0	0	639,473	177,301	1,385,192
Hypertensive disease	0	0	0	2,560,562	1,063,073	4,205,132
Lung cancer	1,346,579	529,951	2,062,912	2,703,748	1,142,155	4,148,475
Colorectum cancer	15,282,178	6,261,097	23,936,360	20,425,635	7,844,742	31,467,857
