# Supplementary material for: Avoidable diet-related deaths and cost-of-illness with culturally optimized modifications in diet: The case of Brazil
Source: PLoS One. 2023 Jul 11;18(7):e0288471. doi: 10.1371/journal.pone.0288471 (PMC10335669; doi:10.1371/journal.pone.0288471)
Supplement: Attachment — Submitted filename: Chulaporn comment.docx [file pone.0288471.s003.docx]

This study looked at optimized diet components that were accounted for food prices and preferences and estimated the number of deaths and economic burden and their cost saving from the health system. The methodology is very crucial for credibility of the results. The estimation of these important results for policy decision requires complex approached. However, the approaches were not well described in the current status of the manuscript, especially the attributable cost. In general, important parameters put into the modelling and their corresponding data sources should be listed in the manuscript. The authors often provided the reference of the particular methods used and this leads to insufficient knowledge that readers could earn.

Page 2, line 9, 10: This requires adding more references. (i.e., There was only reference no. 9.)

9	Several studies have performed diet optimization to design diets attaining nutritional goals
10	while respecting other aspects of the diets, such as food preferences and cost constraints.

Page 5, line 21-22: According to the sentence below, the number of sample, number of food subtypes that were diet risk factors, and observed nutrient components of each age-sex subgroup should be presented.

1591 Food items—115 Food subtypes---26 Food subtypes that were diet risk factors (Table 3)----food quantities (Table 3), energy intake and nutrient component (Table 4)

21	Mean food intakes were obtained for each age-sex group and used as starting points to design
22	optimized diets using linear programming models. Twelve age-sex subgroups were defined: < 30y, 31-
23	39y, 41-49y, 51-59y, 61-69, and >=70, all age groups stratified by sex. The Brazilian Food Composition
24	database (13) was used to obtain nutrient content in both observed and optimized diets. Nutrient
25	composition of foods clustered from food subtypes (e.g. different types of rice into ‘rice’) was obtained
26	as the mean composition of the food subtypes weighted by their frequency of reporting in the NDS.

Page 6, line 18, 19: Spell out for LP and “minimized” should be “minimize”

18	xxxxxxx. The objective function in the LP models was defined to minimized the sum of
19	the absolute difference between each optimized and baseline food quantity, described as follows:

Page 8: Table 1: Please check mmHG should be “mmHg”

Page 8, line 2: Add the abbreviation for PUFAs, and mmHG should be “mmHg”

Page 9: Table 2: In Table 2, please check the unit and consistency with Table 1 and line 7, and the correctness of line 7. For sodium in Table 4, the unit was mg. In addition, the unit per day should be labeled.

7 a percentage in relation to the baseline, in grams for fats and milligrams for cholesterol and sodium.

Page 10, line 25: The calculation of attributable death was based on “outcome-risk pair and age-sex group.” Therefore, the results of these should be shown in the manuscript.

Page 10, line 9-11: How was the Trans Fat Macrosimulation used in this study? Please provide more explanation

9	xxxxxxxxxxx model and the Trans Fat Macrosimulation Model is a comparative risk
10	assessment methodology suitable to estimate the health impact of changes in the behavioral risk factors
11	for non-communicable diseases on population and age-sex specific NCD mortality.

Page 11, line 12-15: Please provide brief explanation of the comparative risk assessment macrosimulation model and its use/parameters to calculate the attributable cost.

12	The attributable costs were estimated through a comparative risk assessment macrosimulation
13	model to estimate the proportion of direct costs to the Brazilian National Health System
14	(hospitalizations) and indirect costs related to premature deaths in each diet scenario, expanded from a
15	costing tool developed for hypertension and related cardiovascular disease (16).

Page 11, line 15-18: “Sugar was never been stated before in the method section about how were they related to fruit or vegetable”

15	xxxxxxxxxxxxxxxxxxxxxxxxxxxxxxxxxxxxxxxxxxxxxxxx. The estimation of the
16	direct and indirect costs related to dietary risks (salt, fats, sugars, fruits, vegetables, and fibers) applied
17	the same PAFs related to each health outcome in each age-sex group for each counterfactual scenario
18	as estimated previously using the PRIME.

Page 12, line 8-9: only the relative risks were varied. Were there other parameters that were also varied for sensitivity analysis.

8	Uncertainty intervals are calculated based on 5,000 Monte Carlo iterations which allows the
9	relative risks to randomly vary according to the distribution described in the literature.

Which software was used for each calculation?

Page 12, line 17-19: 1) Spell out for PUFA; 2) All results of each age sex group pertaining this issue should be presented in the Table so that the contents in these lines can be understood.

17	constraints was necessary. Cholesterol constraint was not attained in 70y+ Females; sodium constraint
18	was not attained in 50-60y Females; PUFA constraints were not attained in 30-40y Males and 60y+
19	Females. In all these cases, the constraints were set to be equal or higher than the baseline mean intakes.

Table 3 and Table 4: The unit per day should be labeled.

Page 15, Table 4: Please provide the abbreviation list for Cho., MUFAs and PUFAs

Page 16, line 1-2: Spell out MUFA and PUFA. However, MUFA was not stated in Table 1.

1	fibers, followed by the changes in fats (lowering total, saturated and trans fats, and cholesterol, while
2	increasing MUFA and PUFA) and salt.

Page 16, Table 5: According to Table 1, could the results be broken down by the individual diseases?

The format of the references must be conformed with the journal requirement.

The availability of the data: The summary of important parameters should be presented in the manuscript.

Yes - all data are fully available without restriction (The datasets analyzed during the current study are available on the Brazilian Institute of Geography and Statistics (IBGE) website, [https://www.ibge.gov.br/en/statistics/social/population/25610-pof-2017-2018-pof en.html?=&t=o-que-e]. Variable names, description, and contents are in Portuguese. The Excel file used for running the PRIME is available on <https://www.euro.who.int/en/health-topics/disease> prevention/tobacco/publications/2019/ncdprime-modelling-the-impact-of-national policies-on-noncommunicable-disease-ncd-mortality-using-prime-a-policy-scenario modelling-tool-2019. SAS codes used for diet optimization are available from the corresponding author on reasonable request.)
