# Supplementary material for: Avoidable diet-related deaths and cost-of-illness with culturally optimized modifications in diet: The case of Brazil
Source: PLoS One. 2023 Jul 11;18(7):e0288471. doi: 10.1371/journal.pone.0288471 (PMC10335669; doi:10.1371/journal.pone.0288471)
Supplement: Attachment — Submitted filename: answer sheet.docx [file pone.0288471.s006.docx]

Additional Editor Comments:

Please authors, kindly address the minor concerns. Please also re-check the grammar of text throughout, to sharpen it further ok.

Answer: thank you very much. We carefully revised the manuscript thoroughly (grammar and other unobserved typos). We also identified and corrected few inconsistencies between the results in the text and tables.

Thank you for the opportunity to review the revised manuscript. The author has thoroughly revised the manuscript in accordance with the comments from the reviewers in the first round and also added relevant information in supplementary material.

Minor concern is about typing error.

Example:

Page 5: Line 20: From this list, the items were aggregated if they were different type, different cooking method, or different meat cut of the same food or (e.g., different types of orange into 21 “orange”, different beef cuts into “beef”, etc.).

Table 4: Charbohydrates (%Kcal)

Answer: thank you very much for observing these mistakes. We carefully revised the manuscript thoroughly (grammar and other unobserved typos).
