# Supplementary material for: Avoidable diet-related deaths and cost-of-illness with culturally optimized modifications in diet: The case of Brazil
Source: PLoS One. 2023 Jul 11;18(7):e0288471. doi: 10.1371/journal.pone.0288471 (PMC10335669; doi:10.1371/journal.pone.0288471)
Supplement: Attachment — Submitted filename: Reviewer comment Plos One April 1 2023.docx [file pone.0288471.s002.docx]

**Overall summary**

This is an interesting study. The study evaluated the impact of change in dietary risk factors on health outcomes and economic burden related to noncommunicable chronic diseases in Brazil by setting the goal at optimized diet. This study used linear programming model to design healthier diet and introduced both dietary constraints and food acceptability constraints into the model. The data sources were based on the national survey. The paper is generally well structured and provided valuable information however there are some points that should be added more explanation.

Method section:

1. Is the study sample for baseline dietary intakes equal to 20,112 households (from NDS)?
2. The total number of study sample and the total number of food items of each age-sex subgroup should be provided in Supplementary section.
3. The final list of food items in each age-sex subgroup ranged from 112 to 119. The author should explain why the total number of food items shown in Table 3 was 26.
4. In Diet modeling section (line 26), the author should add more explanation about how many food items were put into the linear programming model and how to select the food items that were put into the model.
5. The topic of Dietary constraints and Food acceptability constraints should be the subtopic of Diet modeling.
6. In Dietary constraint section, the author should add more details about how to develop a set of key healthy dietary component. The food items that are considered as the dietary risks for NCD based on PRIME model are selected (as shown in Table 2), isn’t it? In addition, the author should add more explanation about the differences among the five scenarios, whether it is gradually increased the nutritional profile of diet and which scenario that were assumed to provide the maximum benefit on health outcome.
7. Table 1 should be moved to Supplementary section.
8. In Attributable death section, the author should add more details about how TFA macrosimulation model were used for calculation attributable death. Why was it used as complementary to PRIME model?
9. In Attributable cost section, comparative risk assessment macrosimulation model refer to TFA macrosimulation model, isn’t it?
10. In Attributable cost section, line 16, why was sugar also considered?
11. In Attributable cost section, line 22, the author should provide how much the data from the Brazilian National Health System represent hospitalizations of NCD patients in Brazil.
12. The author should add the reference for discount rate (2% per year).

Result section:

13. In Table 5 and 6, the author should show the data by NCD diseases such as preventable mortality and hospitalization among patients with CHD, stroke, cancer.
14. Unit of preventable deaths should be clarified in Table 5 (per day or per year). In addition, Page 16, line 13-15, the author should add more explanation about how to calculate the number of deaths would be avoided, for example, 0.023 refer to….
15. In Table 6, trend of saving costs in hospitalizations should be similar to trends of preventable death (Table 5) and saving costs of productivity loss (Table 7). Why saving costs in hospitalization in Scenario #4 was equal to zero?

Other issues required minor change:

- In Table 1, head row in column #1, “Risk factor” should be replaced by “Dietary risk factor”.
- In Table 1, the values in the fourth column for saturated fat, PUFAs, Dietary cholesterol, salt is regression parameter.
- In Table 1, unit of change of dietary cholesterol should be 1 mg/day.
- In Table 2, the author should add the quantities of each dietary item as per day or per…
- In Table 3, order of food items was based on type of food, isn’t it?
- In Table 3, why FV (fruits and vegetables) were grouped?
- In Table 4, there were two type of data, the first part presented total Kcal (nutrient intake) and %Kcal from each nutrient and the second part presented mg or g (nutrient content). The author should add the topic such as nutrient intake and nutrient content in each part. In addition, why sum of %Kcal from each nutrient is approximately 128.66%, which is greater than 100%?

References:

- Introduction section, please add reference in Line 23-24 page 3, line 10 page 4.
- The author should format the references according to the guideline of the journal.

Typing error:

- In Discussion section, page 18, line 5 (0,02/day), line 8 (1,03% and 4,63%), the comma used in the numbers should be revised.
- In Discussion section, page 21, the word “under-reporting” and underreporting” should be consistent.
