# Supplementary material for: Avoidable diet-related deaths and cost-of-illness with culturally optimized modifications in diet: The case of Brazil
Source: PLoS One. 2023 Jul 11;18(7):e0288471. doi: 10.1371/journal.pone.0288471 (PMC10335669; doi:10.1371/journal.pone.0288471)
Supplement: Attachment — Submitted filename: Reviewer comment Plos One Round 2 June 19 2023.docx [file pone.0288471.s005.docx]

Thank you for the opportunity to review the revised manuscript. The author has thoroughly revised the manuscript in accordance with the comments from the reviewers in the first round and also added relevant information in supplementary material.

Minor concern is about typing error.

Example:

Page 5: Line 20: From this list, the items were aggregated if they were different type, different cooking method, or different meat cut of the same food or (e.g., different types of orange into 21 “orange”, different beef cuts into “beef”, etc.).

Table 4: Charbohydrates (%Kcal)
